# Evaluation of a community intervention program in Japan using Framingham risk score and estimated 10-year coronary heart disease risk as outcome variables: a non-randomized controlled trial

**DOI:** 10.1186/1471-2458-13-219

**Published:** 2013-03-11

**Authors:** Bing Zhu, Yasuo Haruyama, Takashi Muto, Akiko Yamasaki, Fumiko Tarumi

**Affiliations:** 1 Department of Public Health, Dokkyo Medical University School of Medicine, Tochigi, Japan; 2 Soka City Health Center, Soka, Saitama, Japan; 3 Division of Health Education, Anhui Provincial Center for Disease Control and Prevention, Hefei, China

**Keywords:** Coronary disease, Lifestyle, Prevention, Risk factors

## Abstract

**Background:**

Community-based programs are being widely adopted in the struggle to prevent cardiovascular diseases. No study has been conducted in Japan to evaluate the effects of a community-based health promotion program by using the Framingham risk score and 10-year CHD risk as outcome variables. The aim of the present study was to assess the effects of a program involving 6-month intervention and 18-month follow-up using such outcomes.

**Methods:**

Participants (n = 1,983, 39.5% women, mean age 63.4 years) were selected for the study in 2008. Of these 1,983, 347 (42.4% women) subjects received the 6-month intervention. The intervention included individual counseling and group sessions, among others. After 18 months, 1,278 participants (intervention group: 238, control group: 1,040) were followed up. Changes in the Framingham risk score and 10-year coronary heart disease (CHD) risk were evaluated. ANCOVA and multiple logistic models adjusted for baseline value, age, sex and intervention times were used.

**Results:**

The results showed that the differences in the Framingham risk score and mean 10-year CHD risk were significant in the intervention group compared with the control group after 6-month follow-up (-0.46 and -1.12, respectively) and were also significant after 18-month follow-up (-0.39 and -0.85, respectively). The proportion of those with intermediate 10-year CHD risk (> = 10%) was significantly lower at 6 months (OR 0.30, 95% CI 0.12-0.74) and at 18 months (OR 0.41, 95% CI 0.19-0.92).

**Conclusions:**

The six-month intervention program effectively decreased estimated 10-year CHD risk and the effects were still present at 18-month follow-up.

**Trial registration:**

UMIN-CTR: UMIN000008163

## Background

Morbidity and mortality due to cardiovascular disease are now the leading public health problems in industrialized countries, including Japan [1,2]. Heart disease is the second leading cause of mortality in Japan [2].

Community-based programs are being widely adopted in the struggle to prevent cardiovascular diseases. Community approaches to cardiovascular disease (CVD) prevention are attractive, since they can target all groups in the community and, if effective, may achieve widespread behavioral change and risk reduction. A number of community CVD prevention programs have been implemented over the last 40 years [3].

As the incidence of cardiovascular disease is largely explained by modifiable risk factors (serum cholesterol and reduced high-density-lipoprotein cholesterol, blood pressure and cigarette smoking), reducing risk factors through health promotion focusing on lifestyle is a logical way of preventing disease [4].

In the meantime, as evidence-based health promotion is becoming more and more important, outcome evaluation variables are needed by experts to evaluate the effect of health promotion programs. Since it is not appropriate to trace the incidence of cardiovascular disease for an intervention study, estimation equations to evaluate the incidence rate have been widely adopted. The Framingham risk score used to evaluate 10-year coronary heart disease (CHD) risk is the most popular estimation equation, which was derived from the Framingham Heart Study [5-8]. Many intervention studies implemented in Western countries have used the Framingham risk score to evaluate the effect of intervention studies [9-15].

In Asia, although there has been some controversy over whether the Framingham risk score overestimates the risk of CHD in Asian populations [16,17], it is still used by experts to provide useful information on future CHD events [18,19].

To our knowledge, this study is the first to use the Framingham risk score and 10-year CHD risk to evaluate the effects of a community-based health promotion program in Japan.

The hypothesis in this study was that, similar to studies conducted in Western countries, lifestyle interventions could reduce the Framingham risk score and estimated 10-year CHD risk after 18-month follow-up relative to a control group.

The aim of the present study was to assess the effect of a program involving six-month intervention and 18-month follow-up in a Japanese community using Framingham risk score and 10-year CHD risk as outcome variables.

## Methods

### Study design

The Ministry of Health, Labour and Welfare (MHLW) in Japan started the “Specific Health Check-up Project” nationally in 2008. This study was one part of the project, which was implemented in Soka City in Saitama Prefecture, Japan, with a population of about 233,000 [20,21].

The present study employed a non-randomized controlled trial design. The program was provided from 2008 to 2010, including a 6-month intervention program and an 18-month follow-up program.

### Participants

**Figure 1:**
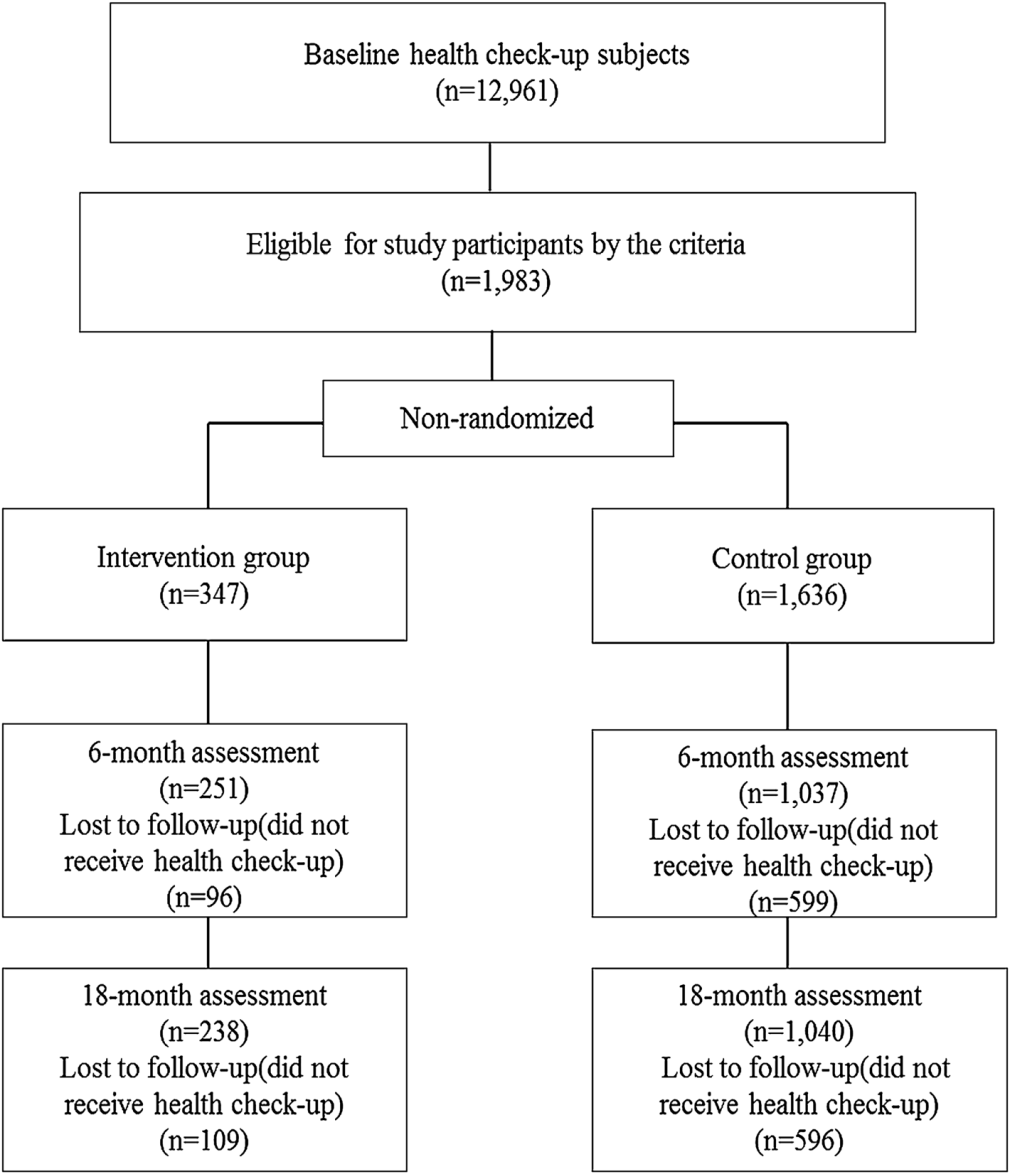
Flowchart of the study protocol.

About 50,000 residents aged from 40–74 were invited to receive a health check-up. As shown in Figure 1, 12,961 residents aged 40–75 years old underwent health check-ups in 2008 and completed the baseline lifestyle questionnaire. Of these 12,961 subjects, 1,983 were selected for the study according to the following inclusion criteria [22], which were separated into two steps: (1) waist circumferences of the subjects as follows: ①waist circumference > =85 cm for males,> = 90 cm for females; ②waist circumference < 85 cm for males, <90 cm for females, and body mass index (BMI) > =25 kg/m^2^; (2) at least one of the following: hemoglobin (HbA1c) >5.2% (JDS, Japan Diabetes Society) (equal to >5.6% [NGSP, National Glycohemoglobin Standardization Program]) [23] or taking diabetes medication; triglycerides (TG) >150 mg/dl or high-density-lipoprotein cholesterol (HDL-C) <40 mg/dl or taking lipid-lowering medication; systolic blood pressure (SBP) > =130 mmHg or diastolic blood pressure (DBP) > =85 mmHg or taking hypertension medication; and having a history of smoking. The subjects who were taking diabetes, lipid-lowering and hypertension medication and were diagnosed as hypertension (SBP/DBP > = 140/90 mmHg), hyperlipidemia (LDL > =140 mg/dl or HDL <40 mg/dl or TG > = 150 mg/dl), diabetes (Fasting blood glucose > = 126 mg/dl or HbA1c > = 6.1% (JDS) (equal to > =6.5% [NGSP])) were advised to be seen by physicians, and were withdrawn from this study. The 1,983 subjects were informed about the program by direct mail and were then allocated into either the intervention or the control group according to the participants’ desire. The numbers of subjects in the intervention and control groups were 347(200 males and 147 females) and 1,636 (999 males and 637 females), respectively. After 6-month follow-up, 1,288 (251 in the intervention group and 1,037 in the control group) participants underwent the second health check-up and completed the lifestyle questionnaire. Finally, after the 18-month follow-up, 1,278 participants (238 in the intervention group and 1,040 in the control group) completed the final health check-up and lifestyle questionnaire in 2010 (Figure 1).

### Risk factor measurements

All of the measurements were provided by medical institutions. Body weight and height were measured with no shoes and excess clothing removed on the same calibrated scale at the baseline, 6-month and 18-month follow-up. BMI was calculated as body weight (kg) divided by the square of the height (m^2^). Waist circumference was measured by nurses. SBP and DBP were measured using auto-manometers (Omron Co., Tokyo, Japan). Fasting blood samples from all subjects were obtained and TG, low-density lipoprotein cholesterol (LDL-C), HDL-C and HbA1c were measured at a laboratory (Saitama, Japan).

Subjects with overweight were defined as having a BMI > =25 kg/m^2^; subjects with hypertension risk were defined as having at least one of the following: SBP > =130 mmHg or DBP > =85 mmHg; subjects with dyslipidemia risk were defined as having at least one of the following: HDL-C <40 mg/dl, LDL-C > =140 mg/dl or TG > =150 mg/dl; subjects with diabetes risk were defined as having a HbA1c > 5.2% (JDS) (equal to >5.6% [NGSP]).

We used the Framingham risk score, which is based on age, TC, HDL-C, SBP and current smoking status, in order to establish risk scores [8]. HDL-C was classified into 4 levels (> = 60, 50–59, 40–49, <40 mg/dl). SBP was classified into 5 levels (<120,120-129,130-139,140-159,> = 160 mmHg). TC was calculated according to the Friedewald equation [24]. Estimated 10-year CHD risk was evaluated according to the Framingham risk score [8]. Moreover, we also used intermediate 10-year CHD risk with a definition of > =10% [8].

### Assessment of lifestyle variables

Information on lifestyle factors such as smoking, drinking alcohol, dietary behaviors and physical activity, medical history and sleeping was obtained by a self-administered questionnaire at the baseline, 6 and 18 months.

Current smokers were defined as those who had been smoking for 6 months or had smoked over 100 cigarettes and were still smoking in the previous month. Drinking alcohol was indicated by the frequency of drinking and the amount of alcohol consumption per day. Dietary behaviors included eating speed, usual time for eating supper, eating snacks and skipping breakfast. Physical activity included regular exercise, daily physical activity and walking speed. Sleeping status was categorized as well or not well.

In this study, the preferable lifestyle behaviors were defined as follows: no smoking, exercise over 30 minutes and 2 times per week, walking or having physical activity over 1 hour every day, walking faster than their peers, not eating fast, not eating dinner less than 2 hours before sleeping, not eating snacks over 3 times every week, not skipping breakfast over 3 times every week, not drinking alcohol every day, drinking alcohol less than 22 g and sleeping well.

### Intervention

All subjects in the intervention and control groups were given 3 health checkups and 3 lifestyle surveys at the baseline, 6-month follow-up, and 18-month follow-up.

The common program consisted of a lecture regarding the purpose of health promotion at the baseline and newsletters featuring general health information provided by local community health workers during the 18-month period.

For the intervention group, a comprehensive program consisting of a 6-month intervention was provided. The 6-month intervention program included individual counseling and group sessions, in addition to the common program.

### Individual counseling

Individual counseling was conducted on the basis of the results of health check-ups and health assessment charts on lifestyle at the baseline (60 minutes per person).

### Group sessions

The group sessions focused on nutrition and exercise (a total of 12 times of 60–120 minutes duration). The nutrition group sessions included talks, a lecture, cooking demonstrations and motivational interviewing. The exercise group sessions included aerobic exercises, stretching, walking and other exercises in a gym or local health or community center. The same program was provided during the daytime and at night for the convenience of participants. Through these approaches, participants were encouraged to set their own goals for lifestyle modification.

### Other interventions by telephone and letter

The other interventions included contact by telephone (a total of 3 times of 5–15 minutes duration) and letter (a total of 3 times). Through telephone calls and letters, subjects were advised to attend the group sessions and motivated to change their lifestyle behaviors. Those who were absent from the group sessions were also followed up by telephone or letter to inquire about the reason. The changes in lifestyle behaviors among the subjects were also evaluated by questions over the telephone.

### Statistical analysis

We selected lifestyle behaviors and cardiovascular disease risks as outcome measures in this study. Lifestyle behaviors included smoking, dietary behaviors, physical activity, drinking alcohol and sleeping. Cardiovascular disease risks included weight, BMI, waist circumference, SBP, DBP, HDL-C, LDL-C, TG and HbA1c.

Baseline cardiovascular disease risk and lifestyle behavior differences were analyzed by Student’s t-test for continuous variables, Mann–Whitney test for nonparametric variables and Chi-squared test for categorical variables.

The mean differences in changes in cardiovascular disease risk factors at 6 and 18 months between the intervention and control groups were assessed by covariance analysis adjusted for the baseline value, age, sex and intervention times. Comparisons of lifestyle behaviors at 6 and 18 months between the intervention and control groups were conducted by multiple logistic regression analysis adjusted for the baseline category, age and sex. The odds ratios (OR) and 95% confidence interval (95% CI) were used to indicate the relative risk ratio between the intervention and control groups. In addition, we determined the probability of risk in subjects with overweight, hypertension risk, dyslipidemia risk, diabetes risk, metabolic syndrome and 10-year CHD risk by multiple logistic regression analysis adjusted for the baseline category, age, sex and intervention times. All statistical analyses were performed using an assumed type I error rate of 0.05. Statistical analyses were performed using SPSS Statistics 20.0 for Windows (SPSS Japan Inc., Tokyo, Japan).

### Ethical consideration

Ethical approval was given by the ethics committee at Dokkyo Medical University (No2057).

## Results

### Baseline characteristics of participants

The baseline characteristics of the study participants are presented in Table 1. The mean age in the intervention group was significantly higher than that in the control group (P < 0.001). In addition, the baseline characteristics of the completed and drop-out subjects in the intervention group were compared (not shown in Table). In 2009, the mean age in the drop-out group was younger than that in the completed group (P = 0.006). In 2010, weight and mean 10-year CHD risk were higher and HDL-C was lower in the drop-out group than in the completed group (P = 0.012, 0.018 and 0.011 respectively). Other variables including the Framingham risk score showed no significant differences between the two groups.

**Table 1:** Characteristics of baseline cardiovascular disease risks and lifestyle behaviors between intervention and control groups

	**Intervention N = 347**	**Control N = 1,636**	**P-value**^**a**^
	Mean ± S.D.	Mean ± S.D.	
Age, year	65.0 ± 7.8	63.0 ± 8.8	<0.001
Weight, kg	66.1 ± 8.1	66.8 ± 9.2	0.166
BMI, kg/m^2^	25.8 ± 2.3	25.8 ± 2.4	0.960
Waist, cm	91.0 ± 5.8	90.9 ± 6.2	0.942
SBP, mmHg	134.0 ± 14.6	135.5 ± 16.6	0.121
DBP, mmHg	79.7 ± 9.7	80.8 ± 10.5	0.077
HDL-C, mg/dl	57.3 ± 14.5	56.2 ± 14.9	0.179
LDL-C, mg/dl	134.0 ± 29.6	133.8 ± 32.1	0.933
TG, mg/dl	151.1 ± 95.8	165.8 ± 130.7	0.287^b^
HbA_1c_,%	5.34 ± 0.47	5.38 ± 0.82	0.395
Framingham risk score, score	14.0 ± 2.5	13.7 ± 2.9	0.085
Mean 10-year CHD risk,%	9.3 ± 6.0	9.4 ± 6.4	0.646
	N (%)	N (%)	
Sex			
male	200 (57.6)	999 (61.1)	0.236
female	147 (42.4)	637 (38.9)	
Current smoker	62 (17.9)	384 (23.5)	0.023
Physical activity			
Exercise over than 30 min, > = 2 times/week	159 (51.6)	595 (39.7)	<0.001
Walking or physical activity over than 1 hour every day	168 (54.9)	707 (47.3)	0.015
Walking faster than their peers	171 (55.9)	771 (52.1)	0.231
Dietary behavior			
Eating fast	88 (28.5)	410 (27.4)	0.686
Eating dinner less than 2 hours before sleeping	62 (20.1)	406 (27.2)	0.009
Eating snacks over than 3 times every week	42 (13.6)	200 (13.4)	0.900
Skipping breakfast over than 3 times every week	22 (7.1)	218 (14.6)	<0.001
Drinking alcohol every day	156 (50.5)	669 (44.7)	0.063
Drinking alcohol less than 22 g	143 (60.9)	666 (56.8)	0.249
Sleeping well	247 (81.0)	1148 (77.2)	0.147

BMI = body mass index, waist = waist circumference, SBP = systolic blood pressure, DBP = diastolic blood pressure, HDL-C = high-density lipoprotein cholesterol, LDL-C = low-density lipoprotein cholesterol, TG = Triglycerides, HbA1c = Hemoglobin, CHD = coronary heart disease.

^a^by chi-squared or Student’s t-test.

^b^by Mann–Whitney test.

The proportions of those with current smoking (P = 0.023), eating dinner late (P = 0.009) and skipping breakfast (P < 0.001) were significantly lower in the intervention group than those in the control group. The proportions of those with regular exercise (P < 0.001) and daily physical activity (P = 0.015) were significantly higher in the intervention group than those in the control group. There were no other significant differences in baseline characteristics between the two groups.

The number of interventions in the intervention group was an average of 7.1 (range from1 to15) (not show in Table).

### Changes in lifestyle behaviors

Table 2 shows the odds ratios of preferable lifestyle behaviors in the intervention group versus the control group. Multiple logistic regression analysis adjusted for each baseline category, age and sex showed that the proportion of those performing regular exercise was significantly higher in the intervention group than in the control group at 6 months (OR 1.79, 95% CI 1.24-2.59). The proportion of those who walked or did physical activity over 1 hour every day was significantly higher in the intervention group than in the control group at 6 months (OR 1.51, 95% CI 1.06-2.14). The proportion of those who walked faster than their peers was significantly higher in the intervention group than in the control group at 6 months (OR 1.45, 95% CI 1.01-2.09). The proportion of those who ate snacks over than 3 times every week was significantly lower in the intervention group than in the control group at 6 months (OR 0.35, 95% CI 0.17-0.71) and it was still lower at 18 months (OR 0.48, 95% CI 0.23-0.98). There were no other significant differences in lifestyle behaviors between the two groups. Moreover, in 2009, 37 people (6 in the intervention group and 31 in the control group) stopped smoking after 6-month follow-up, which accounted for 2.9%; in 2010, 58 people (9 in the intervention group and 49 in the control group) stopped smoking after 18-month follow-up, which accounted for 4.5%.

**Table 2:** Comparison of changes in lifestyle behavior between intervention and control groups

	**At 6 months 251 vs. 1,037**		**At 18 months 238 vs. 1,040**	
	**OR**^**a**^	**95%CI**	**OR**^**a**^	**95%CI**
Current smoker	0.83	0.37-1.87	0.62	0.30-1.28
Physical activity				
Exercise over 30 min, > = 2 times/week	1.79	1.24-2.59	1.27	0.90-1.80
Walking or physical activity over than 1 hour every day	1.51	1.06-2.14	1.30	0.93-1.82
Walking faster than their peers	1.45	1.01-2.09	1.06	0.74-1.52
Dietary behavior				
Eating fast	1.45	0.91-2.30	0.98	0.61-1.57
Eating dinner less than 2 hours before sleeping	0.82	0.52-1.29	0.68	0.43-1.09
Eating snacks over than 3 times every week	0.35	0.17-0.71	0.48	0.23-0.98
Skipping breakfast over than 3 times every week	0.45	0.18-1.09	0.42	0.16-1.15
Drinking alcohol every day	0.78	0.42-1.45	1.23	0.78-1.94
Drinking alcohol less than 22 g	1.57	0.92-2.68	1.28	0.77-2.15
Sleeping well	1.01	0.65-1.56	0.88	0.57-1.35

^a^by multiple logistic regression analysis adjusted for baseline category, age and sex.

### Changes in cardiovascular disease risks

Baseline to 6-month changes in cardiovascular disease risks between the two groups are displayed in Table 3. Presented below and in the tables are the unadjusted means and adjusted P values by using covariance analysis controlling for age, sex, baseline values and intervention times.

**Table 3:** Comparison of changes in cardiovascular disease risk between intervention and control groups at 6-month follow-up

		**N**	**Baseline**	**6-month**	**Intervention vs. Control**	
			**Mean ± S.D.**	**Mean ± S.D.**	**Difference**	**P-value**^**a**^
Weight, kg	Intervention	251	65.6 ± 8.0	63.7 ± 8.2	-0.93	0.002
	Control	1037	65.7 ± 8.3	65.2 ± 8.6		
BMI, kg/m^2^	Intervention	251	25.8 ± 2.2	25.1 ± 2.3	-0.37	0.004
	Control	1037	25.6 ± 2.2	25.4 ± 2.4		
Waist, cm	Intervention	250	90.9 ± 5.9	88.5 ± 6.6	-0.77	0.129
	Control	1036	90.3 ± 5.6	89.6 ± 6.3		
SBP, mmHg	Intervention	251	133.7 ± 14.0	130.8 ± 13.0	-1.77	0.212
	Control	1037	134.8 ± 15.5	133.0 ± 15.0		
DBP, mmHg	Intervention	251	80.0 ± 9.4	77.4 ± 8.7	-1.54	0.092
	Control	1036	80.4 ± 10.3	79.3 ± 9.7		
HDL-C, mg/dl	Intervention	251	57.9 ± 15.2	58.5 ± 15.1	0.91	0.267
	Control	1037	56.8 ± 15.1	56.6 ± 15.3		
LDL-C, mg/dl	Intervention	250	134.7 ± 30.1	130. ± 30.3	2.78	0.227
	Control	1036	131.9 ± 30.9	129.4 ± 29.7		
TG, mg/dl	Intervention	251	141.1 ± 65.8	130.8 ± 71.0	-14.9	0.150
	Control	1036	158.8 ± 125.9	148.2 ± 114.3		
HbA_1c_,%	Intervention	251	5.33 ± 0.43	5.25 ± 0.38	-0.05	0.179
	Control	1033	5.33 ± 0.62	5.33 ± 0.57		
Framingham risk score, score	Intervention	250	14.0 ± 2.4	13.9 ± 2.4	-0.46	0.002
	Control	1029	13.9 ± 2.7	13.9 ± 2.9		
Mean 10-year CHD risk, %	Intervention	250	9.1 ± 5.9	8.9 ± 6.0	-1.12	0.001
	Control	1030	9.4 ± 6.2	9.6 ± 6.5		

BMI = body mass index, waist = waist circumference, SBP = systolic blood pressure, DBP = diastolic blood pressure, HDL-C = high-density lipoprotein cholesterol, LDL-C = low-density lipoprotein cholesterol, TG = Triglycerides, HbA1c = Hemoglobin, CHD = coronary heart disease.

^a^by covariance analysis adjusted for baseline values, age, sex and intervention times.

The mean decreases in weight (P = 0.002) and BMI (P = 0.004) in the intervention group showed significant differences compared with those in the control group. The average Framingham risk score in the intervention group showed a significant decrease (P = 0.002). The mean 10-year CHD risk also showed a significant decrease in the intervention group (P = 0.001).

HDL-C and LDL-C in the intervention group showed slight increases compared with those in the control group, but the increases were not statistically significant. All other cardiovascular disease risks showed a decrease in the intervention group, but the reductions were not statistically significant.

The changes in cardiovascular disease risks between the two groups at 18 months are shown in Table 4. Covariance analysis adjusted for baseline values, age, sex and intervention times showed that the average weight (P = 0.003), BMI (P = 0.007), waist circumference (P = 0.039) and TG (P = 0.021) in the intervention group were significantly decreased at 18 months. The Framingham risk score (P = 0.022) and mean 10-year CHD risk (P = 0.027) were significantly decreased in the intervention group compared with those in the control group. DBP, HDL-C and LDL-C in the intervention group showed slight increases compared with those in the control group, but the increases were not statistically significant. All other cardiovascular disease risks showed a decrease in the intervention group, but the reductions were not statistically significant.

**Table 4:** Comparison of changes in cardiovascular disease risk between intervention and control groups at 18-month follow-up

		**N**	**Baseline**	**18-month**	**Intervention vs. Control**	
			**Mean ± S.D.**	**Mean ± S.D.**	**Difference**	**P-value**^**a**^
Weight, kg	Intervention	238	65.4 ± 7.9	63.8 ± 8.2	-1.08	0.003
	Control	1040	66.0 ± 8.7	65.4 ± 9.2		
BMI, kg/m^2^	Intervention	238	25.7 ± 2.2	25.1 ± 2.3	-0.38	0.007
	Control	1040	25.7 ± 2.3	25.5 ± 2.6		
Waist, cm	Intervention	238	90.8 ± 5.7	88.9 ± 6.5	-1.13	0.039
	Control	1040	90.6 ± 5.9	90.0 ± 6.7		
SBP, mmHg	Intervention	238	133.9 ± 14.5	131.2 ± 14.0	-0.91	0.520
	Control	1040	135.2 ± 16.1	132.4 ± 14.8		
DBP, mmHg	Intervention	238	79.6 ± 9.6	78.1 ± 9.0	0.27	0.778
	Control	1040	80.7 ± 10.5	78.4 ± 9.9		
HDL-C, mg/dl	Intervention	238	58.6 ± 15.0	58.6 ± 14.8	1.10	0.214
	Control	1040	56.6 ± 15.1	56.3 ± 15.3		
LDL-C, mg/dl	Intervention	238	134.2 ± 29.1	127.0 ± 27.9	0.59	0.823
	Control	1040	133.9 ± 31.5	127.5 ± 31.7		
TG, mg/dl	Intervention	238	143.6 ± 74.2	136.5 ± 76.2	-25.1	0.021
	Control	1040	163.4 ± 131.1	156.7 ± 119.4		
HbA_1c_,%	Intervention	238	5.32 ± 0.39	5.31 ± 0.39	-0.03	0.429
	Control	1040	5.35 ± 0.66	5.38 ± 0.57		
Framingham risk score, score	Intervention	238	13.9 ± 2.4	14.0 ± 2.5	-0.39	0.022
	Control	1040	13.9 ± 2.7	14.1 ± 2.9		
Mean 10-year CHD risk,%	Intervention	238	8.7 ± 5.8	8.8 ± 5.9	-0.85	0.027
	Control	1040	9.3 ± 6.4	9.8 ± 6.6		

BMI = body mass index, waist = waist circumference, SBP = systolic blood pressure, DBP = diastolic blood pressure, HDL-C = high-density lipoprotein cholesterol, LDL-C = low-density lipoprotein cholesterol, TG = Triglycerides, HbA1c = Hemoglobin, CHD = coronary heart disease.

^a^by covariance analysis adjusted for baseline values, age, sex and intervention times.

Table 5 shows the odds ratios of cardiovascular disease risks in the intervention group versus the control group. Multiple logistic regression analysis adjusted for each baseline category, age, sex and intervention times showed that the proportion of those with intermediate 10-year CHD risk (> = 10%) was significantly lower in the intervention group than in the control group at 6 months (OR 0.30, 95% CI 0.12-0.74) and at 18 months (OR 0.41, 95% CI 0.19-0.92). The proportion of those with dyslipidemia risk was significantly lower in the intervention group than in the control group at 18 months (OR 0.59, 95% CI 0.36-0.97).

**Table 5:** Odds ratios of cardiovascular risks in intervention versus control group at each risk with baseline

	**At 6 months 251 vs. 1,037**		**At 18 months 238 vs. 1,040**	
	**OR**^**a**^	**95%CI**	**OR**^**a**^	**95%CI**
Overweight	0.62	0.34-1.10	0.56	0.31-1.01
Hypertension risk	1.06	0.66-1.70	0.79	0.49-1.27
Dyslipidemia risk	0.75	0.45-1.24	0.59	0.36-0.97
Diabetes risk	0.71	0.35-1.46	0.65	0.32-1.29
Intermediate 10-year CHD risk (> = 10%)	0.30	0.12-0.74	0.41	0.19-0.92

CHD = coronary heart disease.

^a^by multiple logistic regression analysis adjusted for baseline category, age, sex and intervention times.

## Discussion

The major finding of the present study is a significant decrease in the mean 10-year CHD risk at 6-month follow-up and that the effects were still sustained at 18-month follow-up. The number of subjects with intermediate 10-year CHD risk (> = 10%) also decreased significantly at each follow-up time.

A previous study also showed significant change in 10-year CHD risk after lifestyle intervention. The results from the PREMIER Trial showed that, in individuals with prehypertension or stage 1 hypertension, 2 multicomponent behavioral interventions (EST + DASH and EST) significantly reduced the estimated 10-year CHD risk by 12% and 14%, respectively [9]. The DEPLOY pilot study indicated that community-based delivery of the DPP lifestyle intervention could have a significant effect on prevention of CHD in overweight adults with abnormal glucose metabolism. At 4 and 12 months, the intervention group experienced significant decreases in 10-year risk from baseline (-3.28%, P < 0.001; and -2.23%, P = 0.037) compared with control subjects (-0.78%, P = 0.339; and +1.88%, P = 0.073) [14]. The California WISEWOMAN Project reported that the improvement in the 10-year CHD risk was greater for an enhanced intervention group (EIG) than for a usual care group (UCG), and this improvement was significantly greater when the women’s CHD risk levels were in the upper quartile at baseline [15].

From the results of our study, other cardiovascular disease risk factors did not show significant results, except for weight and BMI. This may have been caused by the 10-year CHD risk reflecting the comprehensive effect of intervention. Maybe some of the cardiovascular disease risk factors changed a little, but this was still not significant. When we combined the changes in cardiovascular disease risk factors together into the 10-year CHD risk, it showed a significant change.

The present study also showed the sustainment of the effect when the intervention was stopped after 18-month follow-up. This proved that the effect of a short-term intervention can be sustained after a long-term follow-up period, greater than one year.

Similar results were also shown in other previous studies. Lindstrom et al. found that lifestyle intervention in people at high risk for type 2 diabetes resulted in sustained lifestyle changes, a modest difference in body weight change and reduction in diabetes incidence, which remained after the individual lifestyle counseling stopped [25]. A study conducted by Elmer et al. showed that, over 18 months, persons with prehypertension and stage 1 hypertension could sustain multiple lifestyle modifications that improve control of blood pressure and could reduce the risk for chronic disease [26]. Four-year results of the Look AHEAD Trial indicated that intensive lifestyle intervention can produce sustained weight loss and improvements in other CVD risk factors in individuals with type 2 diabetes [27].

The results of the present study indicated significant decrease in weight and body mass index at 6-month follow-up. In addition, the reductions were sustained and showed significantly stronger decrease at 18-month follow-up. This proved that, by implementing lifestyle intervention, weight loss can be achieved, which is similar to the results of previous studies.

In a lifestyle intervention study implemented among persons at high risk for cardiovascular disease and diabetes in a rural community, 52% of participants met the 7% weight loss goal and 66% achieved at least a 5% weight loss [28]. However, the sample size was very small (N = 84 and N = 65, respectively). A study conducted by Lindstrom et al. in 2003 indicated that, after 1 and 3 years, weight reductions were 4.5 and 3.5 kg in the intervention group and 1.0 and 0.9 kg in the control group, respectively [29]. In the present study, weight reduction was 1.5 kg in the intervention group and 0.6 kg in the control group after 18 months.

The present study has limitations. Firstly, our study has a non-randomized study design. The participants were allocated to either the intervention or the control group on the basis of each participant’s desire. The subjects with a strong desire to improve their lifestyle were more inclined to accept the intervention and then acquired a better effect of changes in cardiovascular disease risk and lifestyle behavior. However, since the baseline cardiovascular disease risk factors were similar in the two groups, the non-randomized study design may not affect the main significant results in the changes of cardiovascular disease risk in our study.

Secondly, the assessment of lifestyle behavior change in the study was based on a self-reported questionnaire. This may have resulted in some recall bias when evaluating the change in lifestyle behavior and have led to little significant change in lifestyle behavior between the intervention and control groups. Further study should focus on a detailed method to evaluate the change in lifestyle behavior, such as using pedometers to measure change in physical activity.

Thirdly, this study has a high drop-out rate. On the one hand, as the results showed, younger people were more inclined to withdraw from our study, maybe because they were not interested in the form of the intervention. For example, younger people may prefer to choose more vigorous exercises instead of the jogging and gymnastics done in our study. On the other hand, subjects with higher BMI, thus having higher 10-year CHD risk, were more inclined to withdraw from our study, which limited the significance of the results.

Lastly, the number of participants was relatively small, especially in the intervention group, which might have limited the significance of the results and the generalization to the middle-aged Japanese population. Regarding the generalization of this study, the intensity of intervention that targeted a large percentage of the population who are at high risk of CVD may have been too strong. In a future study, a new method of intervention of less intensity should be developed, which can be applied worldwide, considering the limited ability of health services in many areas.

## Conclusions

Our study demonstrates that the six-month intervention program effectively improved the cardiovascular disease risk and estimated 10-year CHD risk. Moreover, the effects were still present at the 18-month follow-up.

## Consent

Written informed consent was obtained from the patient for publication of this report and any accompanying images.

## Competing interests

The authors declare that they have no competing interests.

## Authors’ contributions

BZ coordinated data collection, carried out analysis and drafted the manuscript. YH conceived the study, participated in the study design, coordinated and participated in data collection and critically reviewed the manuscript. TM conceived the study, participated in the study design, and critically reviewed the manuscript. AY and FT conceived the study, were involved in the study design and participated in data collection. All authors read and approved the final manuscript.

## Pre-publication history

The pre-publication history for this paper can be accessed here:

http://www.biomedcentral.com/1471-2458/13/219/prepub
